# Recent Progress in Obstetrics and Gynecology in Germany

**Published:** 1888-02

**Authors:** E. S. McKee

**Affiliations:** Cincinnati, Ohio


### RECENT PROGRESS IN OBSTETRICS AND GYNECOLOGY IN GERMANY.

BY E. S. MCKEE, M. D., CINCINNATI, OHIO.

Salpingitis has been divided by Sanger into five different divisions. These are: 1. Salpingitis gonorrhœica, produced by the gonococcus of Neiser. 2. Salpingitis tuberculosis, produced by the bacillus tuberculosis of Koch. 3. Salpingitis actinomy cotica, produced by the actinomy bovis of Bollinger. 4. Salpingitis septica. 5. Salpingitis syphilitica.

Iodoform gauze has been found of great value by Fritch recently in the palliative treatment of carcinoma, which he terms the dry method. It relieves the foul discharges, hemorrhage and pain so that the patients think they are well. He has also used it with success after removing sloughing portions of the placenta or intra-uterine polypi.

The diagnosis of beginning carcinoma cervis uteria, always a question of the greatest importance, has gained in meaning recently since the operative treatment of cancer of the uterus has rendered its cure possible, provided the case be recognized at a sufficiently early stage. The known symptoms are numerous, yet, the difficulty of distinguishing the beginning of cancer from erosion will always exist. Stratz has studied this subject thoroughly, and gives the following four important points:

1. The diseased surface is everywhere sharply separated from the sound tissue; it nowhere gradually changes from one to the other.

2. A difference in level between the diseased part and the healthy can always be recognized.

3. The cancerous portions have always a yellowish tint.

4. The malignant parts show small yellowish-white, glistening raised points, at least in certain places.

In the excessive vomiting of pregnancy Crede recommends the administration, every five minutes, of teaspoonful dose of nourish ment, preferably iced milk, the patient lying absolutely quiet and taking the nourishment through a glass tube.

Chazan has reported an interesting case of this comp'aint in which no abnormality could be discovered about the patient. She was, however, inconsolable at the idea ot being pregnant. She was put under ether and made to believe that the fœtus had been removed. The vomiting ceased from that time. This case has led Chazan to believe that, perhaps, in most cases, hyperemesis gravidarum was due to some affection of the nervous system, or of the mind, and not to some abnormality of the genital organs, as some authors believe.

Among many German obstetricians absolute non-interference is the rule in the third stage of labor. The command "hands off" is absolute.

The teachings of Crede are tending towards the entire letting alone of the genitals during labor and the days succeeding it. This distinguished obstetrician, unless some abnormality presents, does not make a vaginal examination at all. He makes his diagnosis entirely by external palpation and manipulation. He teaches that one should, for eight or nine days after labor, neither examine, wash out, nor do anything to the genitals, unless there are positive indications therefor.

Hypnotism, or Syggignocism, a means of doing away with the pains of labor introduced by Pritzel, of Vienna, seems to be gaining some followers among those who possess the required power.

The question of antiseptics may be fairly stated as follows: It is the practice of the majority to disinfect the hands with a 1.1000 solution of corrosive sublimate. External genitals, 1.2000; vagina or uterine cavity, 1.4000. The vagina and especially the uterine cavity are washed out only on the strongest indications either just after birth or during confinement to bed. The amount used to irrigate those cavities is about 2 litres. In post partum hemorrhage of the uterus from atony a solution of 1.3000 is used. The sublimate solution is considered as contra-indicated in women suffering from anæmia, phthisis, general cachexia or diseases of the kidney and digestive organs; also those having extensive wounds of the vulva or taking mercurial preparations. It is found that vaginal or intra-uterine irrigation is frequently followed by absorption of the injected liquid, especially if its escape be in any way impeded. When this occurs mercury can be quickly detected in the fæces. The solution 1.1000 is injected into the uterus only in severe cases, as tympanites of the uterus, putrefaction of the fœtus in the uterine cavity or septic puerperal fever. Not more than one minute's time is allowed for the injection, which is followed by copious injections of distilled water. In cases where there has been an expulsion of the macerated fœtus, a solution of 4 1000 is used. This is also used in the endometritis consecutive to the expulsion of the fœtus in premature delivery. This solution is of service in puerperal endometritis and accompanied by fetid vaginal discharge, and should be followed by copious injections of water.

For the disinfection of instruments carbolic acid is in general use. Angerer, of Munich, has claimed that the sublimate solution may be rendered permanent in ordinary undistilled water by adding to the water as much (by weight) of common salt as there is present corrosive sublimate.

The following are the rules for the physicians who wish to visit the laparotomies which are performed in the clinic of Olshausen:

1. On the day of the operation not to come in contact with infectious material of any kind.

2. To come to the operation freshly bathed and in clean linen and clothes which have not been worn in the sick room.

3. To touch no instruments, sponges or anything which is used in the operation.

4. To be there promptly at the appointed hour, as at the beginning of the operation the doors will be closed. Such were the rules of Schrœder and are now carried out by Olshausen and Martin. Gusserow is not quite so strict with his visitors.

The question is often asked, How soon after coming in contact with septic material is a physician justified in attending a case of labor? The reply is, in Vienna, as soon as you have time to change your clothes and go through washing with an antiseptic solution of a reliable character.

In the T clinic, Carl Braun's, in the Allgemeine Krankhaus, in Vienna, the assistant has charge of the wards and conducts personally all complicated cases. At the same time he is constantly giving instruction on the cadaver to the students and practitioners taking operative courses. He is often summoned from the operating table in the pathological building to make a forceps delivery. He would remove his coat and proceed to a most careful washing of the hands and arms. He not only washes them, but he scrubs them and scrubs them well, then dips them into a solution of the permanganate of potassium and then into a solution of muriatic acid. In the second clinic the hands are cleansed by a powder consisting of ground kernels and shells of bitter almonds. This powder seems to possess very great cleansing and absorptive properties.

The use of axis traction forceps, according to Carl Braun, would result in the bringing of living children, in many instances, through deformed pelves, where in the absence of these forceps craniotomy would be necessary. He uses quite frequently the Simpson forceps modified by himself, which he terms tri-form forceps. The instrument as modified, Braun thinks, can be used in the low or high operation, and, in fact, any and every possible occasion in which an instrument is indicated.

The application of a four per cent. solution of cocaine to the upper part of the vagina and cervix during dilatation, and to the ostium vaginæ and perineum during the expulsive effort, is followed by good results, preventing pain in some for twenty minutes.

The conservative Cæsarean Section of Sanger is a method which is rapidly gaining ground. From all parts of Germany come reports which are favorable and constantly improving. This continued and increasing success is very gratifying to those who confidently expect this operation to ultimately displace craniotomy in most cases. Leopold, whose statistics are as gratifying probably as those of any one, recommends complete closure of the abdominal cavity by the continued suture after the protrusion of the uterus. The hemorrhage after the cut into the uterus he controls by the rubber tube or manual compression. He takes great care that the uterine cavity is entirely cleared from decidua. He wishes the uterine sutures to be very exact in their coaptation. He stimulates contraction by manual massage of the sutured uterus.

Professor Gusserow, of Berlin, when operating, follows this plan: He commences the excision on three finger breadths below the umbilicus and continues it to three or four inches above the symphisis pubes. The abdomen is opened, the uterus presents and the walls close behind it. The uterus is surrounded in its lower half below the child's head with a rubber tube the size of the finger which is held there. Sutures are passed through the adductor muscles to prevent the protrusion of the intestines, which it will do unless there is much vomiting If the bowels protrude retain them with warm cloths. The uterus is opened with an incision beginning near the fundus and extending down to the inferior uterine segment to the place where the peritoneum is movable. If the placenta lies in the line of the incision a large amount of dark blood will burst forth. Cut through this and the liquor amnii will gush out. Any hemorrhage which may occur from the uterine incision can be controlled by drawing on the rubber band surrounding the uterus. The child is then removed. The uterus generally remains relaxed during the remaider of the operation. The placenta and membranes are removed from the uterus and the cavity is strewn with iodoform. He takes about eight silver sutures to close the incision. These enclose muscle without decidua. About sixteen silk sutures are then applied which penetrate the peritoneum only. Resection of the muscle is sometimes necessary, but not always; if the peritoneum extends over the muscle some distance it is not. If hemorrhage is now present stop it by ligating the spouting arteries. If the uterus still remains relaxed, cause it to retract by applying sponges soaked in hot sublimate solution. Powder the suture line with iodoform, replace the uterus in the abdominal cavity and close this by suture.

Fifty cases of Sanger's operation have been reported so far, with the following results: For the mother, recovery in thirty-six cases, or seventy-two per cent; death in fourteen cases, or twenty-eight per cent. Result for children, born alive, forty-six, or ninety-two per cent.; died four, or eight per cent. Germany had thirty-four with thirty recoveries and four deaths; children, thirty-two living, two dead. Austria five cases with two recoveries and three deaths; children, five living and no deaths. United States, six cases, two recoveries and four deaths; children, four living and two dead. Italy, three cases, two recoveries and one death; children, three alive. Russia, two cases, no recoveries, two deaths; children, two living. France, one case, one recovery and no deaths; one child alive. It is easy to see that Germany is far in the lead. The very best reports come to us from Leipsic and Dresden. Of the seven cases done in Leipsic, there were seven recoveries of mothers and seven living children. Of the fourteen cases in Dresden, thirteen recovered, and fourteen living children were born.

The first fifty cases after Porro's method resulted in twenty-one recoveries, or forty-two per cent. After Sanger the first fifty cases resulted in thirty-eight recoveries, or seventy-two per cent. After Porro, twenty nine died, or fifty eight per cent. After Sanger, fourteen died, or twenty-eight per cent. This shows a difference of thirty per cent. in favor of the conservative method of Sanger.

